# News Items of General Interest

**Published:** 1914-09

**Authors:** 


### News Items of General Interest.

Dr. C. R. Reed, of Philadelphia, has accomplished the feat of substituting an artificial eye for an injured one and enabling the patient to move the artificial eye in perfect synchronism with his good eye. The operation was accomplished by placing a gold sphere in the stocket, stitching the membranes and muscles to it and then fastening the glass eye in the front of this. It remains to be seen if this will prove practical.

According to the State Health Department, 846 persons have lost their lives from typhoid fever in Texas in the past year. This is a shame and a disgrace, and it seems it is time that the doctors of Texas were making a heroic effort to educate the people concerning this disease. With the discovery and successful use of the typhoid vaccine, this appears unpardonable.

In a recent report given out by Governor Colquitt it is stated that there are 490 insane persons in the county jails, with relatives and on the poor farms who should be in the State institutions. How much longer will the State permit, unhindered, the increase of these defectives, and continue to take the money that should go to educating its young for the building and maintenance of this ever-increasing output of mental defectives who should never have been born?

In the entire State the number of physicians has increased only 456 in the past ten years. At present there are 6885, 9 of whom are under 20 years of age. One hundred and sixty-eight of the 6000 are negro doctors.

The War Department has just let the contract to a Chinese firm for the construction of an army hospital at Fort Schaffer, Hawaii.

Dr. R. E. Hall, President of the Houston Dental Society, has recently patented a new dental instrument, which is known as an articulator. This is said to be very superior to the one now in use, which is known as the Gysi articulator. Dr. Hall has sold the patent to the S. S. White Dental Manufacturing Company, of Philadelphia.

The Rice Brothers, of Ward, La., have recently built and equipped a sanatorium for the treatment of pellagra patients. Dr. A. L. Chapman, who has great success in treating pellagra, is in charge of the sanatorium.

---

The following letter from Dr. Kenney is appreciated by the JOURNAL, and will, we believe, be of interest to its readers:

SAVOY HOTEL,
LONDON, August 20, 1914.

*My Dear Mrs. Daniel:*

The two letters that you addressed to me at San Antonio have just reached me. Had heard of Dr. Daniel's death through my brother. It was a shock to me, yet not wholly unexpected. He was very feeble the last time that I saw him. I feel that I have lost a good friend and that the medical profession of Texas has lost one of its best and most noble characters. It always made me feel better to talk to him, and I do not believe that there is a superior writer in the medical profession today. You have my sincere sympathy in your great bereavement. If at any time I can serve you it will be my pleasure to do so. Shall be glad at all times to hear of the progress and prosperity of the TEXAS MEDICAL JOURNAL. It is the duty of the profession of Texas to support an independent journal. Am sure that they will do it and that yours will be the one, for it deserves it.

Have during the past four months visited all of the chief surgical clinics of Europe. When I return home will write a résumé of this trip for the TEXAS MEDICAL JOURNAL, if you can find a place for the article.

Expect to leave next Saturday for New York and will be home about the middle of September, the German Emperor permitting, perhaps I should say.

The horrors of war are now upon this country. One can hear the tread of soldiers night and day. It is more than awful to think of these now prosperous countries going to ruin. They have already been set back ten years.

It was the opinion of a majority of the 1200 surgeons from America attending the recent Congress of Surgeons in this city that the German Emperor is suffering from one stage of paresis.

With kindest personal regards, and wishing you every success with the TEXAS MEDICAL JOURNAL, I am,

Sincerely yours,

JNO. W. KENNEY.

---

Believing that the following letter will prove interesting to Dr. Abbott's host of friends in Texas, we publish it in full:

August 21, 1914.

*Dear Mrs. Daniel:*

Enclosed please find check for $1.00 in payment of a year's subscription to the "Red Back." I wish you much success with it because you are making it a valuable journal. Please accept my hearty thanks for the copies sent me.

I have just finished visiting the food, health and drug officials of thirty-two States studying the law enforced by them, and otherwise getting acquainted with them as a basis for the organization of my office here. I have been very busy organizing my office, and have not had time to think of what has been going on in Texas. I wish you much success, and I trust everything is well with you.

Cordially, your friend,

J. S. ABBOTT,

Chemist in Charge, State Co-operative Food and Drug Control.

---

THE TEXAS MEDICAL JOURNAL will be sent to any reputable physician with the distinct understanding that, if it is carefully read throughout the year and is then thought not to be worth the subscription price, on receipt of notice to that effect, the money will be as cheerfully refunded as it was received.

PHILLIPS'

### Phospho-Muriate of Quinine

COMP.

An Aromatic Syrup Containing Phosphoric Acid and Phosphates of Potash, Magnesia, Lime and Iron with Muriate of Quinine and Strychnine

A Reliable Tonic and Reconstructive

*Free from Alcohol*

### Phillips' Digestible Cocoa

COMP.

*Made from Fine Selected Beans of Cacao Theobroma*

A Delicious Food Beverage
and valuable Substitute for Tea or Coffee

"THE COCOA WITH A RICH CHOCOLATE FLAVOR"

### Change of Address.

Dr. Bert Knight, from Winnsboro to Quitaque.
Dr. Benton, from Greenville to Peniel.
Dr. Curtis, from Blessing to Bay City.
Dr. M. D. Brandenberger, from Mason to Seguin.
Dr. J. T. Boone, from Ballinger to Trickham.
Dr. R. L. Vineyard, from Temple to Amarillo.
Dr. Aug. Streit, from Galveston to Marlin.
Dr. D. S. Speer, from Port Arthur to Sour Lake.
Dr. T. P. Junkin, from Brownwood to Dallas.
Dr. J. T. Saunders from Fort Worth to Zulch.
Dr. W. W. Fowler, from Hamilton to Dallas.
Dr. T. H. Bates, from Houston to Camden.
Dr. J. B. Treon, from Floresville to Poth.
Dr. D. Hinckson, from Fort Worth to Corinth.
Dr. G. C. Kindley, from Galveston to Temple.
Dr. W. P. Delafield, from Mt. Pleasant to Paris.

Dr. E. S. Miller from Ballinger to San Benito.

The Surgical Publishing Company, of Chicago, announce their removal to 30 North Michigan Avenue, Chicago.

